# P-1128. Pilot Cross Sectional Demographic Study of Pediatric Patients with Reported Penicillin Allergy in a Tertiary Rural Regional Health Center

**DOI:** 10.1093/ofid/ofae631.1315

**Published:** 2025-01-29

**Authors:** Kevin Feldhaus, John Richter, Peter Paul Lim, Emily Sass, Hannah Reedstrom, Tobias Meeissner, McKenna Deaton, Michael Borgmann

**Affiliations:** University of South Dakota Sanford School of Medicine, Sioux Falls, South Dakota; University of South Dakota Sanford School of Medicine, Sioux Falls, South Dakota; Avera McKennan University Health Center, Sioux Falls, South Dakota; Avera McKennan Hospital University & Medical Center, Sioux Falls, South Dakota; Avera McKennan, Sioux Falls, South Dakota; Avera Cancer Institute, Sioux Falls, South Dakota; Avera Cancer Institute, Sioux Falls, South Dakota; Avera McKennan Hospital & University Health Center, Sioux Falls, South Dakota

## Abstract

**Background:**

Penicillins remain reliable agents and are considered drugs of choice for select infections.^1-3^ Approximately 10% of the US population report a penicillin allergy with majority of the labels placed in childhood.^4,5^ There is a paucity of data describing the role of socio-demographic factors to understand the epidemiology of pediatric penicillin allergy especially in the rural settings.^6^

**Methods:**

This is a cross sectional retrospective demographic query of pediatric patients 0-18 years old with reported penicillin allergy in the electronic medical record from 2018-2023 in a rural tertiary health system in South Dakota.

**Results:**

Of 350,847 patients, 10,077 (2.9%) were identified with penicillin allergy label. Of which 5,162 (51%) were male. The median age was 11(6,15). There were 8,499 (88%) Caucasians, 432 (4.5%) American Indian/Alaskan Native, 126 (1.3%) Black/African American, 49(0.5%) Asian, and 3(< 0.1%)) Pacific Island/Native Hawaiian. Amoxicillin was the most common reported allergy in 6,738 (67%) patients, penicillin in 3,163 (31%) and Augmentin in 699 (6.9%). Severe reaction was reported in 2,921 (46%) patients with rash being the most common reported reaction in 5,566 (55%). Asthma was identified in 564 (5.6%) patients. Physicians are the primary providers for 5,778 (74%) among which family medicine physicians composed the majority (5,154, 66%). Penicillin allergy labels were recorded by APP/nursing in 7,202 (71.4). Majority of patients had commercial insurance 6,485 (71%) while 2,139 (23%) had government insurance. Patients’ home addresses that could be mapped using the RUCA classification, majority of patients belonged to rural (2,901,37%) and micropolitan (2,867, 37%) areas. Area Deprivation Index median was 66.

**Conclusion:**

Penicillin allergy is most prevalent among Caucasian pediatric patients in our rural health system with Native Americans comprising the majority of other ethnic minorities. Amoxicillin is the most common reported allergy. Heterogeneous severity and characterization of reactions have been reported for these allergies. A small percentage of patients have identified co-morbidities. Socio-demographic factors differ across pediatric patients with penicillin allergies in our cohort with overall ADI suggesting above the national median.

**Disclosures:**

**Tobias Meeissner, PhD**, BioNTech: Stocks/Bonds (Public Company)|Cellworks: Grant/Research Support|GeneDX: Stocks/Bonds (Public Company)|Labcorp: Grant/Research Support|Tempus: Grant/Research Support

